# A Scoping Review of POLG-Related Cerebellar Ataxia: Insights and Clinical Perspectives

**DOI:** 10.5334/tohm.1027

**Published:** 2025-11-10

**Authors:** Stefania Kalampokini, Iraklis Keramidiotis, Stylianos Ravanidis, Piergiorgio Lochner, Vasilios K. Kimiskidis, Georgios M. Hadjigeorgiou

**Affiliations:** 1 First Department of Neurology, Aristotle University of Thessaloniki, AHEPA University Hospital, Stilponos Kyriakidi 1, Thessaloniki, Greece; 2 Department of Neurosurgery, Aristotle University of Thessaloniki, AHEPA University Hospital, Thessaloniki, Greece; 3 ECONCARE, Athens, 11528, Greece; 4 Department of Neurology, Saarland University Medical Center, Homburg, Germany; 5 Medical School, University of Cyprus, Nicosia, Cyprus

**Keywords:** POLG mutation, POLG-related ataxia, cerebellar ataxia, movement disorders, phenotype-genotype

## Abstract

**Background:**

Cerebellar ataxia is one of the most common movement disorders in mitochondrial disease, with *POLG* mutations being a frequent cause. This scoping review aimed to summarize current knowledge regarding cerebellar ataxia due to *POLG* mutations, focusing on epidemiological, clinical, radiological features and genotype-phenotype correlations.

**Methods:**

We searched PubMed and Web of Science databases for all articles published in English till September 2025 describing cases of *POLG*-related cerebellar ataxia.

**Results:**

In homozygous or compound heterozygous *POLG* mutation carriers, cerebellar ataxia seems to be progressive, and can initiate from either the bulbar muscles, trunk, or limbs. Age at onset varies greatly, ranging from birth to the early 70s. The most common variants in *POLG*-related cerebellar ataxia are W748S and A476T, localized in the linker region of *POLG* gene. Cerebellar ataxia due to *POLG* mutations can present in combination with progressive external ophthalmoplegia, sensory neuropathy, epilepsy (including status epilepticus), headache, other hyperkinetic movement disorders such as myoclonus and tremor, cognitive or affective disorders. Brain imaging commonly reveals atrophy of the vermis or cerebellar hemispheres, cortical atrophy, and/or bilateral T2/FLAIR lesions in both white matter and deep brain nuclei, including inferior olivary nuclei.

**Conclusion:**

*POLG-*related ataxia should be included in the differential diagnosis of slowly progressive cerebellar ataxias. *POLG*-related disease comprises a continuum of clinical features; the combination with progressive external ophthalmoplegia, sensory neuropathy, epilepsy, hyperkinetic movement disorders, as well as characteristic imaging findings, can aid the diagnosis of this underdiagnosed entity. These findings contribute to a better characterization of the phenotype-genotype relationship in the extended pool of *POLG*-related mitochondrial diseases.

**Highlights:**

This review summarizes current knowledge regarding cerebellar ataxia due to *POLG* mutations. A slowly progressive cerebellar ataxia in combination with sensory neuropathy, progressive external ophthalmoplegia, epilepsy, myoclonus, and characteristic imaging findings, including cerebellar atrophy, bilateral lesions in deep brain nuclei (thalami, olivary nuclei) should raise suspicion for *POLG*-related disease.

## Introduction

Mitochondria regulate multiple aspects of neuronal function and development due to their role as energy suppliers, producing adenosine triphosphate (ATP) [1]. Disturbance of ATP production leads to a lack of energy, which mainly affects tissues with high energy consumption, such as brain and muscle [2]. The polymerase γ (*POLG*) gene, a nuclear gene located on chromosome 15, encodes the mitochondrial DNA polymerase, an enzyme located within the inner mitochondrial membrane responsible for replication and repair of mitochondrial genome (mtDNA) [3]. POLG is a heterotrimer, comprising a catalytic subunit (encoded by *POLG1* gene) and two smaller identical accessory subunits (encoded by *POLG2* gene) [4]. The catalytic subunit has three functional domains: an amino-terminal 3’-> 5’ exonuclease domain responsible for proofreading (first third of protein), a highly conserved linker domain (center of the protein), and a carboxy-terminal polymerase domain accountable for replication of mitochondrial DNA (last third of the protein) [5]. The accessory subunits are essential for binding and processing of the synthesized mtDNA [4].

Mutations in *POLG* gene can lead to mtDNA instability, mtDNA depletions or multiple mtDNA deletions affecting energy production in neurons [4]. POLG-related disease mainly follows a recessive mode of inheritance, albeit rarely a single allele mutation has been reported [4]. POLG mutations represent the most prevalent single-gene cause of mitochondrial disease [4]. They have been associated with various clinical phenotypes, including ophthalmological, audiologic, endocrine, gastrointestinal, renal, respiratory and neurological disorders, with onset from infancy to late adulthood [4,6]. Moreover, biallelic *POLG* mutations are associated with several epilepsy phenotypes [7,8,9]. Status epilepticus is a frequent manifestation of *POLG*-related disease; it was reported in up to approximately 80% of patients in a large multicenter study, and was the presenting symptom of the disease in up to 40% of patients in the same study [10].

The prevalence of movement disorders in mitochondrial disease ranges from 6.2% to 30% [11,12]. Movement disorders in *POLG*-related disease include myoclonus [7,8,13,14,15], dystonia [7,11,16], chorea [7,13], parkinsonism [11,17], and restless legs syndrome [11]. Ataxia is one of the most common movement disorders associated with mitochondrial disease [12,18], and can manifest as cerebellar, sensory, or combined [18]. *POLG* mutations seem to be a frequent cause of cerebellar ataxia, accounting for up to 11% of cases in whom repeat expansion diseases have been excluded, as reported in a large German cohort [19].

In this scoping review, we summarize current knowledge regarding cerebellar ataxia due to *POLG* mutations, focusing on epidemiological, clinical, radiological features and genotype-phenotype correlations.

## Methods

We conducted a literature search in databases Pubmed and Web of Science from 1978 till September 2025 in the English language, following the PRISMA guidelines for Scoping Reviews [20]. The search strategy was as follows: ((polymerase gamma mutation) OR (POLG mutation)) AND (cerebellar ataxia). The exclusion criteria were articles not in the English language, animal studies, review articles, articles without describing cerebellar ataxia in the phenotype (e.g. articles describing pure sensory ataxia or Sensory Ataxic Neuropathy, Dysarthria, and Ophthalmoparesis syndrome (SANDO), Alpers-Huttenlocher syndrome). The full texts of all eligible articles were reviewed by three independent authors (SK, IK, SR) to assess their relevance to the research question. The references of the retrieved articles were also scanned for relevant articles. The information retrieved from the articles was charted in a data-charting Excel form, jointly developed by two reviewers (SK, SR). This information was number of patients, gender, age of onset, gene mutation, brain MRI finding, neurological (chronic progressive ophthalmoplegia-CPEO, headache, movement disorders, epilepsy, cognitive and psychiatric symptoms, hypacusis) and non-neurological symptoms (liver/gastrointestinal problems, diabetes), clinical characteristics of ataxia (limb ataxia, gait ataxia, nystagmus, dysarthria), outcome/follow-up and laboratory findings (lactate, cerebrospinal fluid findings, muscle biopsy). The quality of eligible studies was evaluated using the Joanna Briggs Institute (JBI) critical appraisal tool [21]. The JBI Checklist for case reports determines the possibility of bias in their design, conduct, and analysis using 8 items. The majority of the included studies were of high quality (7 or higher), and a few (3 studies) were of moderate quality (6 out of 8). The statistical analysis (frequencies of the above-mentioned variables) was conducted with SPSS version 25. Cases from articles containing more than one patient with a *POLG* mutation and cerebellar ataxia were included in the analysis separately, as long as adequate clinical features were provided. X^2^ test was used in the subgroup analysis (<40 or >40 years old), in order to detect differences in the various variables, as well as in the analysis of factors associated with worse prognosis.

## Results

**Figure 1:**
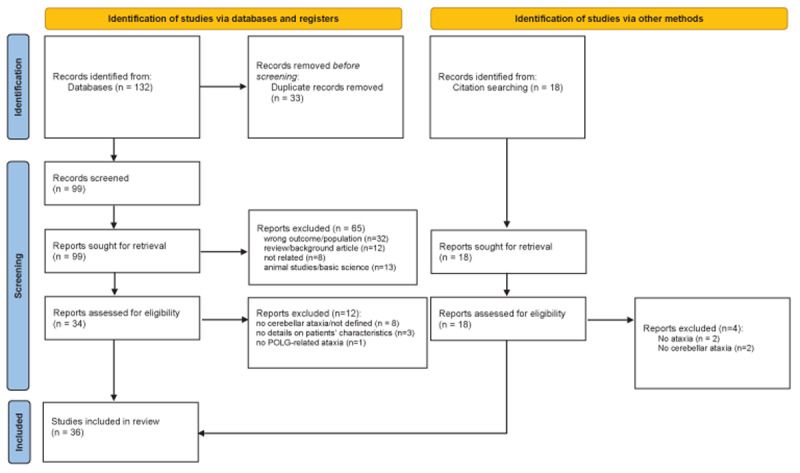
Flow chart of studies included in the scoping literature review. *Source*: Page MJ, et al. BMJ 2021;372:n71. doi: 10.1136/bmj.n71.

The initial search from databases yielded 132 studies, of which 33 were duplicates. From the 99 remaining records, after exclusion of animal studies, review articles, and not related studies or studies with other patient populations based on title/abstract screening, 34 studies were assessed for eligibility (full text was screened). After excluding studies with patients without cerebellar ataxia or without essential information about patients’ characteristics, 22 studies remained for inclusion in the scoping review. In those studies, 14 additional papers from the search of citations were included, bringing the total to 36 studies (Supplementary Table 1). In the statistical descriptive analysis, only the patients having cerebellar ataxia due to POLG mutations were included. The demographic, clinical, and imaging features of patients with POLG-related cerebellar ataxia can be seen in Tables 1 and 2. The flow chart of the included studies can be seen in Figure 1.

**Table 1:** Demographics and common clinical findings of patients with POLG-related cerebellar ataxia (N = 184).


		MEDIAN (RANGE) OR n (%)

**Demographics**		

Age at onset (years)		25 (4–77)

Age at diagnosis (years)		43 (9–80)

Gender	female	100 (54.3%)

	male	55 (29.8%)

	n/a	29 (15.8%)

Pathogenic variant	*W748S (POLG1)*	93 (38.3%)*

	*A467T (POLG1)*	65 (26.7%)*

	*R627Q (POLG1)*	18 (7.4%)*

	*POLG2*	7 (2.9%)*

	*other*	60 (24.7%)*

Zygosity	homozygous	62 (33.7%)*

	heterozygous compound	75 (40.7%)*

	heterozygous (autosomal dominant variant)	10 (5.5%)*

	n/a	37 (20.1%)*

**Clinical findings**		

Ataxia		

gait ataxia	yes	122 (66.3%)

	no	5 (2.7%)

	n/a	57 (31.0%)

dysarthria ± dysphagia	yes	104 (56.5%)

	no	16 (8.7%)

	n/a	64 (34.8%)

limb ataxia	yes	71 (38.6%)

	no	11 (6%)

	n/a	102 (55.4%)

nystagmus	yes	48 (26.1%)

	no	44 (23.9%)

	n/a	92 (50%)

Polyneuropathy	yes	142 (77.2%)

	no	13 (7.1%)

	n/a	29 (15.7%)

CPEO	yes	114 (62%)

	no	53 (28.8%)

	n/a	17 (9.2%)

Epilepsy	yes	80 (43.5%)

	no	69 (37.5%)

	n/a	35 (19%)

Cognitive impairment	yes	87 (47.3%)

	no	63 (34.2%)

	n/a	34 (18.5%)

Psychiatric symptoms	yes	40 (21.7%)

	no	102 (55.4%)

	n/a	42 (22.9%)

Headache	yes	35 (19%)

	no	126 (68.5%)

	n/a	23 (12.4%)

Myoclonus	yes	55 (29.9%)

	no	97 (52.7%)

	n/a	32 (17.4%)

Tremor	yes	31 (16.8%)

	no	112 (60.9%)

	n/a	41 (22.3%)

Outcome/Follow-up	partial improvement**	4 (2.2%)

	persistence of symptoms	14 (7.6%)

	loss of independent walking	6 (3.3%)

	wheelchair-bound	14 (7.6%)

	death	24 (13%)

	n/a	122 (66.3%)


**Abbreviations:** CPEO: chronic progressive ophthalmoplegia, *numbers referring to the total number of alleles reported, **partial improvement referring to improvement of certain symptoms such as seizures or movement disorders.

**Table 2:** Common imaging finding of patients with POLG-related cerebellar ataxia (N = 184).


IMAGING FINDINGS		

Cerebellar atrophy	yes	58 (31.5%)

	no	66 (35.9%)

	n/a	60 (32.6%)

Cerebellar signal changes	yes	43 (23.4%)

	no	76 (41.3%)

	n/a	65 (35.3%)

Cortical changes (atrophy, signal change)	yes	55 (29.9%)

	no	68 (37%)

	n/a	61 (33.1%)

Thalamus signal changes	yes	29 (15.8%)

	no	91 (49.5%)

	n/a	64 (34.7%)

Olivary nucleus signal changes	yes	18 (9.8%)

	no	98 (53.3%)

	n/a	68 (36.9%)

Normal brain MRI	yes	10 (5.4%)

	no	94 (51.1%)

	n/a	80 (43.5%)


**Abbreviations:** MRI: magnetic resonance imaging, n/a: not applicable.

The studies dated from 2004 to 2025. The patients included in the analysis were in total 184, 100 (54.3%) were female, 55 (29.8%) were male, and in 29 (15.8%) gender was not reported. Median age at presentation was 43 years (range 9 to 80 years), and median age of onset was 25 (range 4 to 77 years). Patients with *POLG*-related cerebellar ataxia had a wide variety of symptoms, including gait ataxia reported in 122 (66.3%) of cases, dysarthria with or without dysphagia reported in 104 (56.5%), limb ataxia in 71 (38.6%), and nystagmus in 48 (26.1%). The latency of developing cerebellar ataxia in patients with *POLG* mutations varied significantly, from being the presenting symptom up to 40 years after disease onset (mean 2.9 ± 6.5 years). Ataxia was the initial symptom of presentation in the majority of cases, i.e., in 66 patients (35.9%), followed by epilepsy (34 patients, 18.5%), headache (11 patients, 6%), CPEO (8 patients, 4.4%), while many studies did not report details on initial symptoms (Suppl. Table 2).

Cerebellar ataxia due to POLG mutations presented in combination with other neurological and non-neurological symptoms. Those were in descending frequency neuropathy (reported in 142 patients, 77.2%), progressive external ophthalmoplegia (CPEO) (114 patients, 62%), epilepsy (80 patients, 43.2%), myoclonus (55 patients, 29.9%), cognitive impairment (87 patients, 47.3%), psychiatric symptoms (40 patients, 21.7%) and headache with migraine-like features (35, 19.0%). Polyneuropathy was, in the vast majority of cases, axonal sensory (77 patients, 41.8%), to a lesser extent, axonal sensorimotor (36 patients, 19.6%) and very rarely mixed, i.e. demyelinating and axonal (4 patients, 2.2%) (type was not specified in 56 patients, 30.4%). Patients with *POLG* mutations developed epileptic seizures of all types, i.e., focal with or without impairment of consciousness (reported in 36 patients,19.6%), generalized seizures (19 patients, 10.3%), and/or status epilepticus (30 patients, 16.3%). Cognitive and psychiatric symptoms were relatively commonly reported, with the most common being affective disorders (anxiety and depression) and, very rarely, psychosis in one case [22].

Other less common neurological symptoms, which accompanied cerebellar ataxia at the same time or later on, were muscle weakness (27 patients, 14.7%) and sensorineural hearing loss (reported in 15 patients, 8.2%). Other movement disorders encountered were tremor, most commonly postural or action tremor (reported in 31 patients, 16.8%), chorea/athetosis (23 patients, 12.5%), dystonia (20 patients, 10.9%), and rarely parkinsonism (7 patients, 3.8%). Therefore, hyperkinetic movement disorders were far more common (40.2%) than hypokinetic movement disorders, i.e., parkinsonism (3.8%). Non-neurological features have been reported in a few case series, with the most common being the elevation of liver enzymes and/or gastrointestinal symptoms (reported in 25 patients, 13.6%), and diabetes (11 patients, 6%).

Brain MRI commonly exhibited different degrees of vermis or cerebellar hemisphere atrophy (58 patients, 31.5%), which in most cases was mild to moderate. Bilateral T2/FLAIR lesions in the white matter of cerebellar hemispheres and/or cerebellar peduncles (reported in 43 patients, 23.4%), cerebral hemispheres (55 patients, 29.9%), especially in the occipital lobes, have also been described in POLG patients. T2/FLAIR lesions in deep brain nuclei such as the thalami or dentate nuclei have been reported in 29 patients (15.8%). Lesions in the inferior olivary nuclei have also been reported (18 patients, 9.8%). MRI spectroscopy was reported in a single case and showed a lactate peak in certain frontal, parietal and occipital cortical areas [23].

Cerebellar ataxia was more frequently caused by homozygous (62 cases, 33.7% of total pathogenic variant analysis) or compound heterozygous *POLG1* mutations (75, 40.7%), while heterozygous mutations, i.e., dominant pathogenic variants, were rarely reported (10, 5.5%). The localization of *POLG1* variants was found mainly in the linker subunit of the POLG protein, with the most common being W748S, A467T, and R627Q (*POLG1*) (found in 93 (38.3%), 65 (26.7%), 18 (7.4%) of the total number of alleles reported, respectively) followed by pathogenic variants in the polymerase and exonuclease regions.

Laboratory ancillary examinations, such as lactate in serum or cerebrospinal fluid (CSF), were also not reported in the vast majority of the included studies (Suppl. Table 2). In a large multicenter study of patients with POLG-related disease, lactate was raised in serum in 35% (n = 29/84) and in CSF in 40% (n = 19/47) [6]. On the other hand, another large case series reported that lactate was not consistently elevated, with only occasional patients having elevated levels [8]. Muscle biopsy results were reported in only 11 of the included articles; the presence of ragged-red fibers, COX-negative fibers, and abnormal respiratory chain activities was found in fewer than half of those [6].

Treatment, in the sense of anti-oxidants such as Q10, flavonoids, vitamins, or other nutrients such as carnitine, has been reported in solely three patients with either no improvement or no reported outcome [24,25,26]. Various antiseizure medications were used in patients with epilepsy, including valproic acid in many instances, before establishing the diagnosis of POLG-related disease, which led to deterioration of seizure frequency or even need for liver transplantation [8,27]. Seizure-freedom was very rare, reported solely in one case [28]. Treatment of other movement disorders, such as dystonia or tremor, was also not reported, except for individual cases treated with botulinum toxin injections or trihexyphenidyl with mixed outcomes [24,29]. One patient with parkinsonism received levodopa but without improvement [24]. Outcome or follow-up was not reported in most studies (122 patients, 66.3%). Loss of independent walking was reported in 6 patients (3.3%), wheelchair-bound (within 4–25 years) or with persistence of symptoms were 14 patients (7.6%) respectively. Improvement of certain symptoms (e.g. reduction of seizure frequency, improvement of CPEO or tremor) was only reported in 4 cases (2.2%). Death was reported in 24 cases (13%), mostly due to status epilepticus or multiorgan failure (1–50 years after disease onset) (Suppl. Table 2). In fact, myoclonus (10/16, 62.5% vs 35/126, 27.8%, *p* < 0.05), epilepsy (17/22, 77.3% vs 52/116, 44.8%, *p* < 0.01) and cortical changes i.e. atrophy (12/16, 75% vs 33/96, 34.4%, *p* < 0.01) were associated with negative prognosis i.e. death; the rest of clinical and radiological parameters were non-significant.

### Subgroup analysis

Patients were divided into two groups according to age of onset, i.e., early onset (≤40 years old), and later onset (>40 years old). Patients with an earlier age of onset suffered status epilepticus more frequently (24/67, 35.8% vs 6/41, 14.6%, *p* < 0.05), while patients with later age of onset (>40 years old) had dysarthria (±dysphagia) more frequently (63/67, 94% vs 41/53, 77.4%, *p* < 0.05). In all the other clinical or imaging characteristics, there was no statistically significant difference between the two groups. Patients with an age of onset <40 years had a marginally worse prognosis (bedridden or death) compared to the other group (16/90, 17.8% vs 8/94, 8.5%, *p* = 0.06) (Figure 4).

## Discussion

In this scoping review, we summarized data from studies, mainly case reports or case series, describing patients with cerebellar ataxia due to *POLG* mutations. Movement disorders are common clinical manifestations of mitochondrial diseases, partly due to the high vulnerability of neurons controlling motor circuits, as a result of mitochondrial respiratory dysfunction and energy failure [30]. Cerebellar hypometabolism and dysfunction are viewed as the main pathophysiological mechanisms of movement disorders in mitochondrial disease [12]. Moreover, the substantial two-way communication between the basal ganglia and cerebellum, which form an integrated functional network, seems to be disturbed in mitochondrial diseases exhibiting movement disorders [31].

Ataxia is one of the most common movement disorders in mitochondrial diseases. [12,18] It can be cerebellar, sensory, or combined, but it is rarely present in isolation [18]. Postmortem histology has revealed variable and selective loss of one or more neuronal types among Purkinje cells, olivary and dentate neurons and/or granule cells [12,18,32]. Mitochondrial disease may also affect the afferent fibers from the spinocerebellar tracts [18] or from the inferior olivary nucleus, leading to cerebellar ataxia [33]. In *POLG* mutation carriers, cerebellar ataxia is often progressive and can start either from bulbar muscles, with dysarthria and dysphonia as presenting symptoms, from the trunk, presenting as gait imbalance, or from the limbs, presenting as symmetrical or asymmetrical limb ataxia. It should be noted that irrespective of the site of onset, it is associated with gaze-evoked nystagmus in less than one third of cases. The predominant features of dysarthria consist of altered prosody, articulatory breakdown, including imprecise consonants, vowel distortions, and prolonged phonemes [35]. Patients with POLG-related ataxia can also present with voice tremor [35]. Dysphagia, with deficits being more pronounced when consuming liquids than with solid or puree consistencies, usually appears later on in the course of the disease [35]. Cerebellar ataxia can be the presenting feature of the disease (in approximately one third of cases in the present analysis), or it can manifest decades after the initial symptoms. Ataxia can develop in any age; however, it seems more common in the <12 years onset and 12–40 years onset groups, as shown in a large cohort study [6]. Still, in most cases, ataxia becomes clinically relevant approximately a decade after disease onset, although some cerebellar features, such as dysarthria, can be evident early during diagnosis. Sensory ataxia can also co-occur, as sensory neuropathy is one of the most common accompanying features of POLG cerebellar ataxia. Impaired position and vibration sense, followed by sensory symptoms in glove-stocking distribution (with or without neurophysiological proven neuropathy) are the most common symptoms, while neuropathic pain is rather rare [13].

Regarding progression of *POLG*-related cerebellar ataxia, it is usually slowly progressive over decades and may lead to severe disability, i.e., inability to stand and walk, but again usually within years or one or two decades [7,9]. Although primary mitochondrial ataxias are associated in general with a progressive ataxia course [33,34], in individual cases, there may be a more rapid pattern of progression. In fact, there has been a single case of a 15-year-old female patient presenting with rapidly evolving cerebellar ataxia, showing severe deterioration within 4 months [24]. Another case report described a middle-aged male patient who became wheelchair-bound due to POLG-related ataxia within four years [36]. A Finnish cohort study reported that although progression of the disease varied, occasionally it was quite rapid, necessitating permanent hospitalization before the age of 50 [13].

The most common neurological symptoms in POLG-related cerebellar ataxia, apart from neuropathy, were CPEO and epilepsy. Limitation of eye movements can be seen early in the disease course, but it is more usual for complete ophthalmoplegia to occur as a late, end-stage sign [33]. Epilepsy can be focal or generalized, often refractory to treatment, leading to status epilepticus with poor prognosis. In fact, status epilepticus or its direct sequelae was a common cause of death in cases where the outcome was reported [6,7,8,9,28]. Notably, occipital seizures with visual phenomena and corresponding occipital EEG focus are characteristic of POLG-related disease [8,9,28]. Other movement disorders, such as myoclonus, tremor, dystonia (focal or segmental), and chorea, in descending order, can accompany cerebellar ataxia due to a *POLG* mutation. Myoclonus can be epileptic, i.e., of cortical origin and was found to be associated with worse prognosis i.e. death in our analysis. Tremor can be either postural or kinetic, and less commonly, rest tremor. Interestingly, palatal tremor has been reported in three cases [8,36,37]. Therefore, it seems that it is more likely to encounter hyperkinetic rather than hypokinetic movement disorders alongside POLG-related cerebellar ataxia. Cognitive impairment was another common accompanying feature, which usually occurred years after disease onset and was reported to be rather mild. Other features such as affective symptoms, i.e., depression or anxiety, migraine-like headache, hearing loss, elevation of liver enzymes, and diabetes were also noted.

Neuroimaging features in POLG-related ataxia, as reported in most reviewed studies, include cerebellar atrophy, which is usually mild or moderate. Bilateral T2/FLAIR lesions in the white matter of cerebral hemispheres (especially in the occipital lobes), cerebellar hemispheres, and cerebellar peduncles are also common findings in POLG-related ataxias. Inferior olivary nuclei signal change or enlargement seems to be characteristic of POLG mutations and can be associated with palatal tremor/myoclonus [8,36,37] T2 lesions can also be seen in the deep brain nuclei, particularly the thalami or dentate nuclei in *POLG* mutations [33]. Unspecific white matter lesions and basal ganglia changes can also occur [11,12,38]. General cerebral atrophy and cortical focal lesions manifesting as T2/FLAIR hyperintensities were more prominent in patients with epilepsy [6]. In those patients, MRI can be normal at presentation (even when EEG shows an epileptic focus), but lesions can appear later on, most likely as a direct result of epileptic activity, representing edema and inflammation, and can then resolve [28]. Large cerebellar lesions resembling infarcts are rarer but may be seen in *POLG-1*-related ataxia [39].

Laboratory findings that aid in the diagnosis of mitochondrial ataxia comprise elevated lactic acid, particularly in the cerebrospinal fluid [33,34], although lumbar puncture was not performed or reported in the vast majority of the included studies. Another element in CSF of patients with POLG-related disease was increased protein [6,8,9,14,23,27], while light pleocytosis was evident in only a few cases [9]. Moreover, biochemical, enzymatic, and histopathological evidence of oxidative phosphorylation impairment from muscle biopsies remains an important supportive clue for the diagnosis of a mitochondrial ataxia [34,40]. Notably, only 12 studies reported data from muscle biopsies of patients with POLG-related ataxia [6,8,9,14,23,26,27,41,42,43,44]. Mitochondrial DNA sequencing in combination with targeted NGS panels on nuclear genes, and more recently, whole-exome sequencing (WES), further improves the diagnostic capacity [34]. The less invasive, genetic-first approach based on early WES or whole genome sequencing (WGS) in stratified cases is commonly preferred in recent years [34].

No clear genotype-phenotype correlations are evident for *POLG* mutations; the same mutation can lead to mtDNA deletions, mtDNA depletion, or both, making the prediction of phenotype based on mutations difficult. [4] Recessive mutations in *POLG1* have been associated with a heterogeneous spectrum of neurological and musculoskeletal disorders, all of which include cerebellar, sensory, or mixed ataxia as a cardinal or an additional feature [45]. On the other hand, dominant mutations in *POLG1* are usually associated with adult-onset progressive external ophthalmoplegia phenotypes and variable neurological manifestations such as cerebellar ataxia, extrapyramidal signs, peripheral neuropathy, mental retardation, hypogonadism, and gastrointestinal motility disorders [41,45,46,47]. There are more than 250 pathogenic mutations in *POLG1* affecting five distinct functional modules of the enzyme; the same mutations have been reported in different syndromes, reinforcing the idea of a *POLG1*-related spectrum of diseases [34]. In the reviewed papers, the three most common pathogenic variants were W748, A467T, and R627Q, which accounted in total for approximately 70% of mutant alleles. These variants lead to amino acid substitutions in the spacer region of the *POLG* protein, which result in insufficient polymerase activity and compromised interaction with the accessory subunit and a severe DNA binding defect [48]. The p.W748S mutation was shown to be common in POLG-related ataxias in European populations [13,14,19,36]. Although A467T and W748S are a common cause of ataxia in northern European countries such as Scandinavia, Russia, and Poland, they seem to be rare in the United Kingdom and Italy [41,49,50]. Cerebellar ataxia can also occur in POLG2-related disease, which is very rare. The clinical spectrum of heterozygous POLG2 mutations comprise cerebellar ataxia, seizures, peripheral neuropathy, CPEO, and other movement disorders (tremor, parkinsonism) in adulthood-onset and metabolic abnormalities and seizures in childhood-onset cases [43,51].

**Figure 2:**
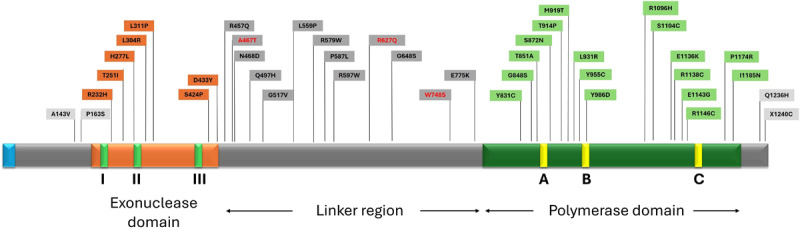
Localization of detected mutations in POLG-protein sequence, most common pathogenic variants can be seen in red color.

Through literature search, we were able to map the localization of all *POLG1* variants in the catalytic subunit that interfere with mitochondrial DNA maintenance [6] and are associated with cerebellar ataxia (Figure 2). The most common pathogenic variants were localized in the linker region, followed closely by those localized in the polymerase and exonuclease regions of the POLG protein [6]. Mutations that reside in the linker region of *POLG* gene have been associated with ataxic and epileptic symptoms [4] with variants W748S and A476T, whether in homozygous or heterozygous state, most commonly reported [16,25,52,53] The possible mechanistic reason behind the effects of mutations in the linker region may lie in severely impaired polymerase activity and disruption of the *POLG2* accessory subunit recruitment, disrupting further binding to DNA and subsequent replication [54]. Pathogenic variants in the polymerase region can lead to various phenotypes [4]; i.e., a heterozygous carrier of p.R1146C presented with severe early-onset cerebellar ataxia [24], while a single p.E1143G (either in homozygous or heterozygous state) was insufficient to cause disease symptoms [47]. The latter mutation may modify symptoms when interacting with an additional heterozygous mutation [8]. Indeed, p.E1143G has been reported in patients with ataxia in combination with mutations in the linker region [8,42]. The integrity of mitochondrial DNA depends on mutations in other nuclear genes as well, such as *PEO1* and *OPA* [55]. Lastly, there has been a case with rapidly progressive cerebellar ataxia within four months in a patient harboring a heterozygous mutation in exon 21 (c.3436C > T, p.R1146C), in the polymerase region [24]. On the contrary, fewer mutations were found in the exonuclease region, which seems to have a limited impact on mitochondrial-related disease progression and leads to milder forms of the disease [4,48].

It has been proposed that the symptoms of *POLG*-related disease differ according to the age of onset, with patients with earlier disease onset having a worse prognosis [6], as also shown in the current analysis for patients with an age of onset earlier than 40 years. Age at onset showed significant variability, ranging from birth to 71 years [6,19,56]. However, in most case series, the age of disease onset was in the 30s or 40s. Age at onset did not seem to be associated with homozygous or heterozygous POLG mutations, although in some case series, the age at onset was significantly earlier in patients with homozygous A467T mutations compared to patients with compound heterozygous mutations [7]. Earlier onset during puberty may be accompanied by systemic involvement of various organs and seizures; later onset during adulthood may result in ataxia, peripheral neuropathy, and seizures [6]. In contrast, even later onset of the disease (over 40 years old) is accompanied by ptosis, progressive external ophthalmoplegia, and ataxia [4,6]. The present analysis showed that patients with earlier age of onset, i.e. younger than 40 years, suffered more frequently status epilepticus, while patients with later age of onset, i.e. older than 40 years old had more frequently dysarthria as a clinical manifestation of ataxia.

**Figure 3:**
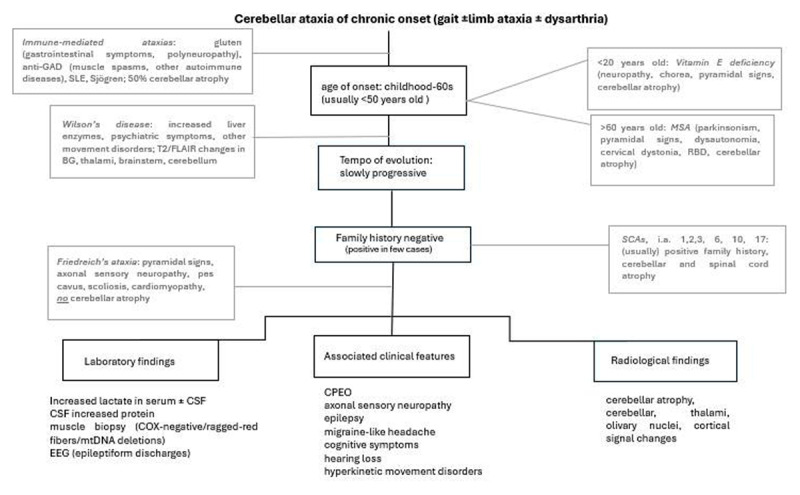
Diagnostic flowchart for POLG-related cerebellar ataxia. Differentials can be seen with light gray color. Figure based on initial figure seen by Wong et al. 2018 [46]. Abbreviations: MSA = multiple system atrophy, GAD = glutamic acid decarboxylase, SLE = systematic lupus erythematosus, SCAs = spinocerebellar ataxias, CSF = cerebrospinal fluid, EEG = electroencephalogram, CPEO = chronic progressive ophthalmoplegia.

POLG-related ataxia shares some common features with Friedreich’s ataxia, such as ataxia with both cerebellar and sensory components, areflexia, impaired vibration sense (in the context of axonal sensory neuropathy), diabetes, and age of onset commonly <40 years [57]. However, some features are distinct to POLG-related ataxia, such as the presence of CPEO, epilepsy (with occipital epileptiform activity), migraine-like headaches, cognitive dysfunction, affective symptoms, hearing loss, and hyperkinetic movement disorders, in particular myoclonus. On the other hand, pyramidal tract signs, square wave jerks, and pes cavus will suggest Friedreich’s ataxia [57], although the latter has been reported in individual cases of POLG-related ataxia as well [13,14,50]. Notably, certain spinocerebellar ataxias (SCAs) include CPEO (SCA 2,3,28,40), epilepsy (SCA 10, 19,22), or neuropathy (SCA 1,2,3,4, 43, 46), as part of their phenotype [58,59]. With regard to features of ataxia, dysarthria with dysphagia seems to be a prevalent feature of POLG-related ataxia [35,60]. Nystagmus has been reported in a few cases that explicitly reported this sign; however, it does not seem to be a ubiquitous finding, such as in spinocerebellar ataxias (SCAs) [61]. Of course, treatable conditions such as immune-mediated ataxias (gluten-related ataxia, anti-GAD-antibodies-associated ataxia, systemic lupus erythematosus, Sjögren’s syndrome) [62,63], or metal storage disorders such as Wilson’s disease [64], which may have an insidious onset, should be ruled out in the early stages of the diagnostic process. Imaging findings of cerebellar atrophy, which is not a feature of Friedreich’s ataxia, in combination with signal changes in cerebellar hemispheres, thalami, (inferior) olivary nuclei, and, less commonly, cortical signal changes, will speak for POLG-related ataxia. On the other hand, SCAs, which are a common differential diagnosis, can present other imaging findings such as brainstem (pontine) or spinal cord atrophy, and the “hot cross bun sign”, which is not exclusively seen in MSA [65,66]. In terms of progression, the progression rate of POLG-related ataxia seems to be faster than Friedreich’s and SCA type 6 but slower than SCA 1, 2, 3, and multiple system atrophy (MSA) [7]. Laboratory findings such as increased lactate in serum and/or CSF, muscle biopsy with ragged red fibers, COX-deficient fibers, and/or multiple mtDNA deletions, when available, are also useful supportive information, although they have low diagnostic sensitivity in POLG-related disease [6]. On the other hand, elevated CSF protein seems to be a more sensitive laboratory diagnostic biomarker [6]. As the age of ataxia onset varies greatly, both pediatric and adult neurologists should be able to suspect POLG-related ataxia. An algorithm as to when to consider a POLG-related ataxia and diagnostic clues to distinguish it from other common ataxias can be seen in Figure 3 and Supplementary Figure 3, respectively.

**Figure 4:**
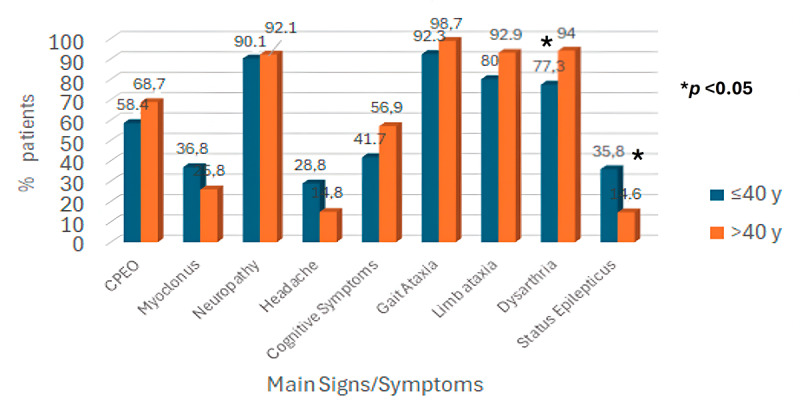
Diagram showing common symptoms of POLG-related cerebellar ataxia, according to age of onset.

We further aimed to document the therapeutic strategies applied in each case study/series. However, most case reports did not describe a therapeutic strategy. Where it was mentioned, most studies employed symptomatic treatment with antiseizure medications, including sodium channel blockers, lamotrigine, topiramate, and even valproic acid, the latter before establishing diagnosis, since its administration is contraindicated in a POLG-related disease [8,14,25,27,29,41,67]. Only three cases reported treatment with different combinations of coenzyme Q10, vitamins C, E, flavonoids, and L-carnitine to enhance mitochondrial function [24,25,26]. Current therapeutic options for patients with *POLG* mutations include mainly nutritional interventions [68] and the application of nucleotides and nucleosides [4]. Considering the former option, a ketogenic diet is thought to stimulate mitochondrial biogenesis, improve mitochondrial function, and lower the burden caused by oxidative stress [69], thus potentially ameliorating the clinical phenotype related to mitochondrial depletion. Additionally, vitamin supplements and/or antioxidants are supposed to support mitochondrial function [4]. Moreover, supplementation with nucleosides (deoxycytidine/dC and deoxythymidine/dT) in a mitochondrial DNA depletion disorder [70] and in *POLG*-deficient fibroblasts has been shown to promote mitochondrial DNA repopulation [70,71].

The present analysis suffers from some limitations, including the underreported or missing data in the included studies, regarding epidemiological, clinical, laboratory, or outcome measures of patients with *POLG*-related ataxia. The studies included were, in the vast majority, case reports or case series. An important issue is the lack of clinical characterization of patients’ symptoms in many cases; particularly, the type of ataxia was often not determined, referring solely to “gait imbalance” or “clumsiness”, and not all common accompanying symptoms of POLG-related disease were reported. Our review indicates a paucity of documentation of these characteristics of patients. Therefore, large multi-center studies are needed in order to fully elucidate cerebellar and extracerebellar characteristics as well as outcome/survival of patients with POLG mutations. Moreover, clinical trials need to consider stratification of mitochondrial diseases not only according to the presenting phenotype but also according to the underlying gene. This is particularly important in light of the availability of genetic testing for these rare disorders and genetic counseling.

To conclude, although *POLG*-related disorders are well-recognized mitochondrial diseases, they are possibly underdiagnosed due to their diverse, overlapping, and late-onset manifestations [72], particularly if standard panels not including *POLG* are used. Mitochondrial ataxias should be included in the differential diagnosis of slowly progressive ataxias. A detailed family history should be collected. In the case of a recessive pattern of inheritance, POLG1-related disorders should be included in the first-line genetic screening for hereditary ataxia. Nonetheless, pathogenic variants in both mtDNA and nuclear DNA can occur *de novo*, manifesting as sporadic ataxias [34]. Helpful features in the diagnosis of POLG-related ataxia include the presence of other hyperkinetic movement disorders such as myoclonus, chorea or dystonia, epilepsy, progressive external ophthalmoplegia, cognitive or affective disorders, and subclinical liver involvement. Neuroimaging in POLG-related ataxias can reveal cerebellar atrophy or T2/FLAIR lesions in the white-matter of cerebral or cerebellar hemispheres, usually bilateral, and in some cases in deep nuclei such as the thalami and inferior olivary nuclei, which can give further diagnostic clues. Therefore, diagnosing a *POLG* mutation-related disorder is challenging and of crucial importance for the improvement of therapeutic management of affected patients. These findings contribute to the better phenotype-genotype characterization of the extended pool of *POLG*-related mitochondrial diseases.

## Additional Files

The additional files for this article can be found as follows:

10.5334/tohm.1027.s1 Supplementary Table 1. Studies of patients with POLG mutations exhibiting cerebellar ataxia.

10.5334/tohm.1027.s2 Supplementary Table 2. Initial symptoms, laboratory findings and outcome of patients in studies of POLG-associated cerebellar ataxia.

10.5334/tohm.1027.s3 Supplementary Table 3. Diagnostic clues to distinguish POLG-related cerebellar ataxia from other common ataxias.
