# Supplementary material for: A Scoping Review of POLG-Related Cerebellar Ataxia: Insights and Clinical Perspectives
Source: Tremor Other Hyperkinet Mov (N Y). 2025 Nov 10;15:55. doi: 10.5334/tohm.1027 (PMC12617407; doi:10.5334/tohm.1027)
Supplement: Supplementary Table 1. — Studies of patients with POLG mutations exhibiting cerebellar ataxia. [file tohm-15-1-1027-s1.pdf]

Author et al, year	N of patients with ataxia	Sex (F/M)	Age of onset ± SD	Genetic profile of POLG mutations	Neurological symptoms				Other symptoms	Imaging findings		
					CPEO	Neuropathy	Movement disorders	Epilepsy		Cerebellar changes	Deep nuclei changes	Cortical changes
**Van Goethem et al, 2004[1]**	1/8	0/1	18	homozygous W748S/ W748S	no	sensory	myoclonus	yes	psychiatric symptoms, intestinal pseudoobstruction	no	no	no
**Winterthun et al, 2005[2]**	6	3/3	14.33 ± 6.19 y.o.	• homozygous A467T/A467T or W748S/W748S (n=4)<br>• c. heterozygotes Q497H/W748S (n=2)	yes (n=4)	axonal (n=2) demyelinating and axonal (n=4)	myoclonus (n=6)<br>tremor (n=2)	yes (n=5)	headache (n=4)<br>cognitive symptoms (n=2)<br>psychiatric symptoms (n=1)	cerebellar atrophy (n=1)<br>cerebellar signal changes (n=3)	olivary nucleus changes (n=1)<br>thalamic changes (n=2)	yes (n=3)
**Hakonen et al, 2005[3]**	14/19	6/8	26.5 ± 10.14 y.o.	• homozygous W748S/ W748S<br>• homozygous E1143G/ E1143G	yes (n=5)	yes (n=13)	myoclonus (n=7)<br>chorea/ athetosis (n=4)<br>tremor (n=7)	yes (n=7)	paresis (n=6)<br>cognitive symptoms (n=9)<br>psychiatric symptoms (n=11)	NR	NR	NR
**Tzoulis et al, 2006[4]**	22/26	15/10	16.46 ± 11.11 y.o.	• homozygous for either A467T/A467T or W748S/W748S (n=18)<br>• c. heterozygotes A467T/W748S (n=7)	yes (n=12)	yes, (n=22, axonal sensorimotor)	myoclonus (n=17)<br>tremor (n=1)	n=18	headache (n=21)<br>cognitive symptoms (n=8)<br>liver involvement (n=10)	cerebellar atrophy (n=4)<br>cerebellar signal changes (n=7)	olivary nucleus changes (n=2)<br>thalamus changes (n=7)	yes (n=13)
**Galassi et al, 2008[5]**	1	1/0	24	homozygous A467T/A467T	yes	axonal sensorimotor	parkinsonism	yes	paresis, psychiatric symptoms	cerebellar atrophy	no	yes (atrophy)
**Harrower et al, 2008[6]**	1	0/1	10	c. heterozygote c.191C>T, c.695G>A, c.2209G>C	no	axonal sensorimotor	tremor	no	paresis	no	no	no
**Paus et al, 2008[7]**	1/2	1/0	28	c. heterozygote A467T/ W748S with E1143G modifier	yes	axonal sensorimotor	myoclonus, chorea/ athetosis	yes	headache, cognitive symptoms	cerebellar atrophy	no	yes (atrophy)
**Schulte et al, 2009[8]**	13/23	12/1	30.15 ± 10.7 y.o.	• homozygous A467T/A467T (n=2)<br>• homozygous W748S/W748S (n=1)<br>• homozygous -10 c>t IVS 16 (n=1)	yes (n=8)	axonal sensory (n=3) axonal sensorimotor (n=5)	no	n=2	paresis (n=3)<br>cognitive symptoms (n=5)<br>psychiatric symptoms	cerebellar atrophy (n=7)	no (n=7), NR (n=6)	no (n=7), NR (n=6)

				• c. heterozygotes A467T/W748S (n=4)<br>• c. heterozygote R627Q/G848S (n=1)<br>• c. heterozygote R627Q/ins A c.3594 (n=1)<br>• c. heterozygote R627Q/R1096H (n=1)<br>• autosomal dominant G517V (n=1)<br>• autosomal dominant N468D (n=1)					(n=5)			
**Schicks et al, 2010**[9]	9	NR	between 12-41 y.o.	• homozygous W748S/W748S (n=2)<br>• homozygous A467T/A467T (n=1)<br>• c. heterozygotes A467T/W748S (n=3)<br>• c. heterozygote R627Q/R1096H (n=1)<br>• c. heterozygote R627Q/G848S (n=1)<br>• c. heterozygote R627Q/insAc.359 (n=1)	yes (n=7)	axonal sensory (n=9)	no	yes (n=3)	paresis (n=3) psychiatric symptoms (n=3)	cerebellar atrophy (n=4)	no	yes (n=1)
**Roshal et al, 2011**[10]	1	1/0	15 y.o.	c. heterozygote A467T/ W748S	no	axonal sensory	no	yes	paresis, headache, cognitive symptoms, liver involvement	cerebellar signal changes	thalamus changes	yes
**Verhoeven et al, 2011**[11]	1	1/0	37 y.o.	heterozygote A736S	no	no	no	no	cognitive symptoms, psychiatric symptoms	cerebellar atrophy	no	no
**Palin et al, 2012**[6]	mother	1/0	35 y.o.	homozygous W748S/W748S with E11436 modifier	yes	yes	no	yes	cognitive symptoms psychiatric symptoms	cerebellar signal changes	no	no
	son	0/1	10 y.o.	homozygous W748S/W748S with	no	yes (axonal	no	yes	psychiatric symptoms	cerebellar signal changes	no	no

				E11436 modifier		sensory)						
**Habek et al, 2012**[12]	1	1/0	13	c. heterozygote A467T/ W748S	yes	sensory	chorea/ athetosis	no	no	cerebellar signal changes	thalamus changes	no
**Hinnell et al, 2012**[13]	1	1/0	childhood	c. heterozygote W748S/T914P	yes	axonal sensory	myoclonus, chorea/ athetosis, dystonia	yes	cognitive symptoms	cerebellar signal changes	thalamus changes	no
**Lax et al, 2012**[14]	1	1/0	20 y.o.	c. heterozygote A467T/ W748S	no	no	myoclonus	yes	cognitive symptoms, psychiatric symptoms, diabetes	NR	NR	yes (ischemic)
**Synofzik et al, 2012**[15]	13	10/3	26.3 ± 10 y.o.	• homozygous for either A467T/ A467T or W748S/W748S (n=9)<br>• c. heterozygotes A467T/ W748S (n=2)<br>• c. heterozygote R627Q / G648S (n=1)<br>• autosomal-dominant Y955C (n=1)	yes (n=13)	axonal sensory (n=12)	myoclonus (23%)<br>chorea (31%)<br>dystonia (31%)<br>parkinsonism (8%)	yes (38%)	cognitive symptoms (69%)<br>liver involvement (8%)<br>diabetes (8%)	cerebellar atrophy (n=9)	thalamus changes (n=3)	yes (n=2)
**Woodbridge et al, 2013**[16]	2/5	1/0	21 y.o.	• c. heterozygote P163S/T851A	yes	sensorimo tor	myoclonus	yes	bowel pseudoobstru ction	no	no	yes
		1/0	15 y.0.	• autosomal dominant Y831C	no	axonal sensory	no	yes	bowel pseudoobstru ction	no	no	no
**Bereu et al, 2016**[17]	15/28	NR	32.86 ± 12.58 y.o.	• homozygous W748S/W748S (n=2)<br>• homozygous W748S/W748S and E1143G/ E1143G (n=1)<br>• c. heterozygote A467T/W748S (n=1)<br>• c. heterozygote A467T/W748S with E1143G (modifier) (n=1)<br>• c. heterozygote A467T/R597W (n=1)	yes (n=14)	sensory (n=13)<br>NR (n=1)	chorea/ athetosis (n=2)<br>dystonia (n=1)<br>parkinsonism (n=1)	yes (n=2)	paresis (n=3)<br>headache (n=6)<br>cognitive symptoms (n=3)<br>psychiatric symptoms (n=5)	cerebellar atrophy (n=4)<br>cerebellar signal changes (n=2)	olivary nucleus changes (n=3)	yes (n=3)

				• c. heterozygote L304R/W748S (n=1)<br>• c. heterozygotes R232H; H277L/T251I; P587L (n=2)<br>• c. heterozygote Y986D/M919T (n=1)<br>• c. heterozygote A467T/R1138C (n=1)<br>• c. heterozygote A467T/L559P (n=1)<br>• c. heterozygote A467T/G848S (n=1)<br>• c. heterozygotes R627Q; Q1236H/ W748S; E1143G (n=2)								
**Van Maldegem et al, 2016[18]**	7	4/3	59.86 ± 8.53 y.o.	heterozygotes c.970-1G>C	yes (n=1)	axonal sensory (n=5) axonal sensorimo tor (n=1)	myoclonus (n=2) dystonia (n=3) tremor (n=7) parkinsonism (n=3)	yes (n=4)	cognitive symptoms (n=5)	cerebellar atrophy (n=7) cerebellar signal changes (n=6)	NR	yes (n=6)
**Janssen et al, 2016[19]**	4/7	2/2	20.75 ± 3.36 y.o.	• homozygous W748S/W748S, E1143G (modifier) (n=1)<br>• homozygous W748S/W748S (n=1)<br>• c. heterozygous A467T/ W748S (n=2)	yes (n=2)	axonal (n=1) axonal sensorimo tor (n=1) axonal sensory (n=1) NR n=1	myoclonus (n=1)	yes (n=4)	cognitive symptoms (n=1) liver malfunction and gastrointestin al disorders (n=4)	cerebellar signal changes (n=1)	no	yes (n=4)
**Nicastro et al, 2016[20]**	1	0/1	50 y.o.	c. heterozygote W748S / I1185N	no	no	palatal tremor	no	diabetes	cerebellar atrophy, cerebellar signal changes	olivary nucleus changes	no
**Henao et al, 2016[21]**	1	1/0	24 y.o.	homozygous W748S/ W748S	yes	sensorimo tor	no	no	no	cerebellar atrophy, cerebellar signal changes	olivary nucleus changes, thalamus changes	no
**Paucar et al,**	1	0/1	48	homozygous	no	axonal	myoclonus,	no	cognitive	cerebellar	no	no

**2016[22]**				W748S/W748S		sensory	chorea/ athetosis, parkinsonism		symptoms	signal changes		
**Mongin et al, 2016[23]**	1	1/0	41	homozygous W748S/W748S	yes	axonal sensory	no	no	no	cerebellar atrophy	olivary nucleus changes	no
**Rossi et al, 2017[24]**	1	1/0	15 y.o.	heterozygote R1146C	yes	no	dystonia	no	no	cerebellar atrophy	no	no
**Vogel et al, 2017[25]**	12/14	9/3	33.08 ± 13.83 y.o.	• homozygous A467T/A467T (n=3)<br>• homozygous W748S/W748S (n=2)<br>• homozygous R627Q/R627Q (n=1)<br>• c. heterozygote A467T/W748S (n=4)<br>• c. heterozygote R627Q/G648S (n=1)<br>• dominant Y955C (n=1)	yes (n=1)	NR	NR	NR	cognitive symptoms (n=9)	NR	NR	NR
**Jerath et al, 2018[26]**	1	0/1	6 y.o.	heterozygote R457Q	NR	sensorimo tor	tremor	yes	paresis, cognitive symptoms	no	no	no
**Piekutowska -Abramczuk et al, 2019[27]**	2/26	1/0	19 y.o.	heterozygote W748S	no	yes (sensorim otor)	no	no	no	cerebellar atrophy	no	no
		0/1	<50 y.o.	heterozygote p.Glu1136Lys	yes	no	no	no	no	NR	NR	NR
**Dosekova et al, 2020[28]**	1	1/0	5	homozygous D433Y	no	axonal sensorimo tor	chorea/ athetosis	no	headache, psychiatric symptoms	cerebellar atrophy	no	yes
**Hikmat et al, 2020[29]**	87 /155	79/76	10 y.o. (median)	41 different POLG variants. The majority (n = 58/83) of patients with early onset disease (<12 years) were c. heterozygote for pathogenic POLG variant. The majority (n= 32/52) of juvenile/ adult-onset disease carried homozygous pathogenic variants	38%	peripheral neuropath y (53%)	no	69%	liver involvement (64%) vomiting (38%) chronic diarrhea (6%)	no	no	54.6%

**Bender et al, 2021**[30]	24	16/8	30.92 ± 15.53 y.o.	• homozygous A467T/ A467T (n=6)<br>• homozygous W748S/ W748S (n=4)<br>• homozygous R627Q/R627Q (n=1)<br>• homozygous W748S/ W748S + E1143G/ E1143G (n=1)<br>• c. heterozygotes A467T/ W748S (n=5)<br>• c. heterozygote R627Q/G848S (n=1)<br>• c. heterozygote A467T/ W748S+E1143G (n=1)<br>• c. heterozygote R627Q/ R1096H (n=1)<br>• c. heterozygote R627Q/ Ins A c.3594c (n=1)<br>• c. heterozygote R627Q/P1174R (n=1)<br>• c. heterozygote P587L/T251I (n=1)<br>• dominant Y955 (n=1)	yes (n=23)	yes (n=11, axonal sensory)	myoclonus (n=10), chorea/athet osis (n=8), dystonia (n=6)	yes (n=8)	diabetes (n=2)	yes (n=10)	olivary nucleus changes (n=4), thalamus changes (n=9)	yes (n=6)
**Nuzhnyi et al, 2021**[31]	11	7/4	29.46 ± 12.2 y.o.	• homozygous W748S/ W748S (n=8)<br>• c. heterozygote W748S/L931R (n=1)<br>• c. heterozygote R579W/L311P (n=1)<br>• c. heterozygote W748S/A143V (n=1)	yes (n=11)	yes (n=10, axonal sensory)	myoclonus (n=3) dystonia (n=3) tremor (n=11)	n=1	cognitive symptoms (n=11) psychiatric symptoms (n=2)	cerebellar signal changes (n=7)	olivary nucleus changes (n=4) thalamus changes (n=4)	yes (n=3)
**Radziwonik et al, 2022**[32]	4	NR	37.75 ± 22.07	• heterozygote S872N (n=1)<br>• heterozygote A467T (n=1)<br>• c. heterozygote A143V/E775K (n=1)<br>• heterozygote T748S (n=1)	yes (n=1)	sensorimo tor (n=2)	myoclonus (n=1)	n=1	hypacusis n=2) headache (n=1) cognitive symptoms (n=1)	cerebellar atrophy (n=2) cerebellar signal changes (n=1)	no	yes (n=1)

**Santos et al, 2022**[33]	1	NR	25 y.o.	homozygous W748S/W748S	yes	yes	no	yes	NR	NR	NR	NR
**Borsche et al, 2024**[34]	2 (siblings)	1/0	30 y.o.	heterozygotes S424P	yes	no	yes (dystonia)	no	no	no	no	no
		0/1	40 y.o.	heterozygotes S424P	no	yes (axonal sensory)	no	no	no	n/a	n/a	n/a
**Smith et al, 2025**[35]	5	1/4	18.5 ± 4.95 y.o. (from 2 patients)	• homozygous A467T/A467T (n=1)<br>• c. heterozygote A467T/X1240C (n=1)<br>• c. heterozygote W748S/R1096C (n=1)<br>• c. heterozygote G848S/S1104C (n=1)<br>• c. heterozygote T251I/P587L/ A467T (n=1)	NR	NR	NR	yes (n=2)	NR	NR	NR	yes (n=2)

Abbreviations: c. heterozygote: compound heterozygote; F/M: females/males; NR: not reported; CPEO: chronic progressive external ophthalmoplegia; y.o.: years old.

Supplementary table 1: Studies of patients with POLG mutations exhibiting ataxia

1. Van Goethem G, Luoma P, Rantamaki M, Al Memar A, Kaakkola S, Hackman P, et al. POLG mutations in neurodegenerative disorders with ataxia but no muscle involvement. *Neurology*. 2004;63(7):1251-7 DOI: 10.1212/01.wnl.0000140494.58732.83.
2. Winterthun S, Ferrari G, He L, Taylor RW, Zeviani M, Turnbull DM, et al. Autosomal recessive mitochondrial ataxic syndrome due to mitochondrial polymerase gamma mutations. *Neurology*. 2005;64(7):1204-8 DOI: 10.1212/01.WNL.0000156516.77696.5A.
3. Hakonen AH, Heiskanen S, Juvonen V, Lappalainen I, Luoma PT, Rantamaki M, et al. Mitochondrial DNA polymerase W748S mutation: a common cause of autosomal recessive ataxia with ancient European origin. *Am J Hum Genet*. 2005;77(3):430-41 DOI: 10.1086/444548.
4. Tzoulis C, Engelsen BA, Telstad W, Aasly J, Zeviani M, Winterthun S, et al. The spectrum of clinical disease caused by the A467T and W748S POLG mutations: a study of 26 cases. *Brain*. 2006;129(Pt 7):1685-92 DOI: 10.1093/braiNRwl097.
5. Galassi G, Lamantea E, Invernizzi F, Tavani F, Pisano I, Ferrero I, et al. Additive effects of POLG1 and ANT1 mutations in a complex encephalomyopathy. *Neuromuscul Disord*. 2008;18(6):465-70 DOI: 10.1016/j.nmd.2008.03.013.
6. Harrower T, Stewart JD, Hudson G, Houlden H, Warner G, O'Donovan DG, et al. POLG1 mutations manifesting as autosomal recessive axonal Charcot-Marie-Tooth disease. *Arch Neurol*. 2008;65(1):133-6 DOI: 10.1001/archneurol.2007.4.

7. Paus S, Zsurka G, Baron M, Deschauer M, Bamberg C, Klockgether T, et al. Apraxia of lid opening mimicking ptosis in compound heterozygosity for A467T and W748S POLG1 mutations. *Mov Disord*. 2008;23(9):1286-8 DOI: 10.1002/mds.22135.
8. Schulte C, Synofzik M, Gasser T, Schols L. Ataxia with ophthalmoplegia or sensory neuropathy is frequently caused by POLG mutations. *Neurology*. 2009;73(11):898-900 DOI: 10.1212/WNL.0b013e3181b78488.
9. Schicks J, Synofzik M, Schulte C, Schols L. POLG, but not PEO1, is a frequent cause of cerebellar ataxia in Central Europe. *Mov Disord*. 2010;25(15):2678-82 DOI: 10.1002/mds.23286.
10. Roshal D, Glosser D, Zangaladze A. Parieto-occipital lobe epilepsy caused by a POLG1 compound heterozygous A467T/W748S genotype. *Epilepsy Behav*. 2011;21(2):206-10 DOI: 10.1016/j.yebeh.2011.03.003.
11. Verhoeven WM, Egger JI, Kremer BP, de Pont BJ, Marcelis CL. Recurrent major depression, ataxia, and cardiomyopathy: association with a novel POLG mutation? *Neuropsychiatr Dis Treat*. 2011;7:293-6 DOI: 10.2147/NDT.S20153.
12. Habek M, Barun B, Adamec I, Mitrovic Z, Ozretic D, Brinar VV. Early-onset ataxia with progressive external ophthalmoplegia associated with POLG mutation: autosomal recessive mitochondrial ataxic syndrome or SANDO? *Neurologist*. 2012;18(5):287-9 DOI: 10.1097/NRL.0b013e318266f5a6.
13. Hinnell C, Haider S, Delamont S, Clough C, Hadzic N, Samuel M. Dystonia in mitochondrial spinocerebellar ataxia and epilepsy syndrome associated with novel recessive POLG mutations. *Mov Disord*. 2012;27(1):162-3 DOI: 10.1002/mds.23960.
14. Lax NZ, Hepplewhite PD, Reeve AK, Nesbitt V, McFarland R, Jaros E, et al. Cerebellar ataxia in patients with mitochondrial DNA disease: a molecular clinicopathological study. *J Neuropathol Exp Neurol*. 2012;71(2):148-61 DOI: 10.1097/NEN.0b013e318244477d.
15. Synofzik M, Srulijes K, Godau J, Berg D, Schols L. Characterizing POLG ataxia: clinics, electrophysiology and imaging. *Cerebellum*. 2012;11(4):1002-11 DOI: 10.1007/s12311-012-0378-2.
16. Woodbridge P, Liang C, Davis RL, Vandebona H, Sue CM. POLG mutations in Australian patients with mitochondrial disease. *Intern Med J*. 2013;43(2):150-6 DOI: 10.1111/j.1445-5994.2012.02847.x.
17. Bereau M, Anheim M, Echaniz-Laguna A, Magot A, Verny C, Goideau-Sevrain M, et al. The wide POLG-related spectrum: An integrated view. *J Neurol Sci*. 2016;368:70-6 DOI: 10.1016/j.jns.2016.06.062.
18. Van Maldergem L, Besse A, De Paepe B, Blakely EL, Appadurai V, Humble MM, et al. POLG2 deficiency causes adult-onset syndromic sensory neuropathy, ataxia and parkinsonism. *Ann Clin Transl Neurol*. 2017;4(1):4-14 DOI: 10.1002/acn3.361.
19. Janssen W, Quaegebeur A, Van Goethem G, Ann L, Smets K, Vandenberghe R, et al. The spectrum of epilepsy caused by POLG mutations. *Acta Neurol Belg*. 2016;116(1):17-25 DOI: 10.1007/s13760-015-0499-8.
20. Nicastro N, Ranza E, Antonarakis SE, Horvath J. Pure Progressive Ataxia and Palatal Tremor (PAPT) Associated with a New Polymerase Gamma (POLG) Mutation. *Cerebellum*. 2016;15(6):829-31 DOI: 10.1007/s12311-015-0749-6.
21. Henao AI, Pira S, Herrera DA, Vargas SA, Montoya J, Castillo M. Characteristic brain MRI findings in ataxia-neuropathy spectrum related to POLG mutation. *Neuroradiol J*. 2016;29(1):46-8 DOI: 10.1177/1971400915621324.

22. Paucar M, Engvall M, Gordon L, Tham E, Synofzik M, Svenningsson P. POLG-Associated Ataxia Presenting as a Fragile X Tremor/Ataxia Phenocopy Syndrome. *Cerebellum*. 2016;15(5):632-5 DOI: 10.1007/s12311-016-0777-x.
23. Mongin M, Delorme C, Lenglet T, Jardel C, Vignal C, Roze E. Progressive Ataxia and Palatal Tremor: Think about POLG Mutations. *Tremor Other Hyperkinet Mov (N Y)*. 2016;6:382 DOI: 10.7916/D86M36RK.
24. Rossi M, Medina Escobar A, Radrizzani M, Tenembaum S, Perandones C, Merello M. Dystonia in a Patient with Autosomal-Dominant Progressive External Ophthalmoplegia Type 1 Caused by Mutation in the POLG Gene. *Mov Disord Clin Pract*. 2017;4(2):266-9 DOI: 10.1002/mdc3.12397.
25. Vogel AP, Rommel N, Oettinger A, Horger M, Krumm P, Kraus EM, et al. Speech and swallowing abnormalities in adults with POLG associated ataxia (POLG-A). *Mitochondrion*. 2017;37:1-7 DOI: 10.1016/j.mito.2017.06.002.
26. Jerath NU, Shy ME. Asymmetric Ataxia, Depression, Memory Loss, Epilepsy, and Axonal Neuropathy Associated with A Heterozygous DNA Polymerase Gamma Variant of Uncertain Significance, c1370G>a (R457Q). *J Neuromuscul Dis*. 2018;5(1):99-104 DOI: 10.3233/JND-170229.
27. Piekutowska-Abramczuk D, Kaliszewska M, Sulek A, Jurkowska N, Oltarzewski M, Jablonska E, et al. The frequency of mitochondrial polymerase gamma related disorders in a large Polish population cohort. *Mitochondrion*. 2019;47:179-87 DOI: 10.1016/j.mito.2018.11.004.
28. Dosekova P, Dubiel A, Karlowicz A, Zietkiewicz S, Rydzanicz M, Habalova V, et al. Whole exome sequencing identifies a homozygous POLG2 missense variant in an adult patient presenting with optic atrophy, movement disorders, premature ovarian failure and mitochondrial DNA depletion. *Eur J Med Genet*. 2020;63(4):103821 DOI: 10.1016/j.ejmg.2019.103821.
29. Hikmat O, Naess K, Engvall M, Klingenberg C, Rasmussen M, Tallaksen CM, et al. Simplifying the clinical classification of polymerase gamma (POLG) disease based on age of onset; studies using a cohort of 155 cases. *J Inherit Metab Dis*. 2020;43(4):726-36 DOI: 10.1002/jimd.12211.
30. Bender F, Timmann D, van de Warrenburg BP, Adarmes-Gomez AD, Bender B, Thieme A, et al. Natural History of Polymerase Gamma-Related Ataxia. *Mov Disord*. 2021;36(11):2642-52 DOI: 10.1002/mds.28713.
31. Nuzhnyi E, Seliverstov Y, Klyushnikov S, Krylova T, Tsygankova P, Bychkov I, et al. POLG-associated ataxias can represent a substantial part of recessive and sporadic ataxias in adults. *Clin Neurol Neurosurg*. 2021;201:106462 DOI: 10.1016/j.clineuro.2020.106462.
32. Radziwonik W, Elert-Dobkowska E, Klimkowicz-Mrowiec A, Ziora-Jakutowicz K, Stepniak I, Zaremba J, et al. Application of a custom NGS gene panel revealed a high diagnostic utility for molecular testing of hereditary ataxias. *J Appl Genet*. 2022;63(3):513-25 DOI: 10.1007/s13353-022-00701-3.
33. Santos M, Damasio J, Carmona S, Neto JL, Dehghani N, Guedes LC, et al. Molecular Characterization of Portuguese Patients with Hereditary Cerebellar Ataxia. *Cells*. 2022;11(6) DOI: 10.3390/cells11060981.
34. Borsche M, Dulovic-Mahlow M, Baumann H, Tunc S, Luth T, Schaake S, et al. POLG2-Linked Mitochondrial Disease: Functional Insights from New Mutation Carriers and Review of the Literature. *Cerebellum*. 2024;23(2):479-88 DOI: 10.1007/s12311-023-01557-x.
35. Smith LA, Olkhova EA, Lax NZ, Ng YS, Taylor RW, Gorman GS, et al. Delineating the mechanisms of cerebellar degeneration in paediatric and adult primary mitochondrial disease. *Acta Neuropathol*. 2025;149(1):53 DOI: 10.1007/s00401-025-02891-6.
