# Supplementary material for: A Scoping Review of POLG-Related Cerebellar Ataxia: Insights and Clinical Perspectives
Source: Tremor Other Hyperkinet Mov (N Y). 2025 Nov 10;15:55. doi: 10.5334/tohm.1027 (PMC12617407; doi:10.5334/tohm.1027)
Supplement: Supplementary Table 2. — Initial symptoms, laboratory findings and outcome of patients in studies of POLG-associated cerebellar ataxia. [file tohm-15-1-1027-s2.pdf]

Author et al, year (number of cases)	Initial symptoms	Latency of ataxia	Laboratory results			Treatment	Outcome/ Follow-up
			Lactate	CSF	Muscle biopsy		
**Van Goethem et al, 2004[1] (n=1)**	epilepsy	5 years	elevated levels	elevated protein content	fibers with subsarcolemmal accumulation of mitochondria containing paracrystalline inclusions	NR	death
**Winterthun et al, 2005[2] (n=6)**	epilepsy (n=2)<br>headache (n=2)<br>ataxia (n=1)<br>headache, ataxia (n=1)	no latency (n=2)<br>3 years (n=1)<br>10 years (n=2)<br>11 years (n=1)	NR	elevated cells and protein content (n=4)<br>normal (n=2)	20% COX deficient fibers (n=1)<br>10% ragged red fibers and COX deficient fibers (n=1)<br>5-10% COX deficient fibers (n=1)<br>normal (n=3)	NR	• death (n=1)<br>• wheelchair-bound (after 20 and 25 years) (n=2)<br>• ataxia (one with seizures) (n=2)<br>• NR (n=1)
**Hakonen et al, 2005[3] (n=14)**	headache (n=1)<br>ataxia (n=4)<br>epilepsy (n=4)<br>neuropathy (n=2)<br>tremor (n=1)<br>ataxia, neuropathy (n=2)	NR (usually presenting symptom)	NR	NR	NR	NR	NR
**Tzoulis et al, 2006[4] (n=22)**	headache (n=7)<br>epilepsy (n=7)<br>ataxia (n=4)<br>headache, epilepsy (n=3)<br>headache, ataxia (n=1)	NR	not consistently elevated, only in few patients	elevated protein levels in a few patients	cytochrome oxidase negative fibers (n=10)<br>normal (n=8)	sodium valproate,<br>two patients underwent liver transplantation	• death (n=6, 2/6 from multiorgan failure)<br>• NR (n=16)
**Galassi et al, 2008[5] (n=1)**	CPEO,<br>weakness of neck and proximal limb muscles	NR	normal levels	elevated protein levels	ragged-red and cytochrome-c-oxidase negative fibers	valproate for 6 months	bedridden
**Harrower et al, 2008[6] (n=1)**	ataxia	12 years	NR	elevated protein levels	NR	NR	wheelchair-bound (after 25 years)
**Paus et al, 2008[7] (n=1)**	headache,<br>cognitive decline,<br>epilepsy	4 years	NR	NR	mild unspecific myopathological alterations	botulinum toxin into both upper eyelids	Improvement of CPEO
**Schulte et al, 2009[8] (n=13)**	NR	NR	NR	NR	NR	NR	NR
**Schicks et al, 2010[9] (n=9)**	NR	NR	NR	NR	NR	NR	NR
**Roshal et al, 2011[10]**	epilepsy	no latency	normal levels	NR	normal	oxcarbazepine, levetiracetam, zonisamide,	seizures

**(n=1)**						topiramate,<br>valproic acid,<br>phenytoin and 10% dextrose intravenously with L-carnitine and coenzyme Q-10	
**Verhoeven et al, 2011[11] (n=1)**	ataxia	NR	normal levels	NR	NR	clomipramine, citalopram	ataxia
**Palin et al, 2012[6] (n=2)**	ataxia (n=2)	no latency	NR	NR	few COX negative fibers (n=2)	NR	• death (after 20 years) (n=1)<br>• NR (n=1)
**Habek et al, 2012[12] (n=1)**	ataxia	10 years	normal levels	NR	NR	vitamin C 1 g qid,<br>coenzyme Q10 100 mg tid,<br>L-carnitine 500 mg qid,<br>lamotrigine 25 mg bid	NR
**Hinnell et al, 2012[13] (n=1)**	epilepsy	1 year	normal levels	normal levels	normal	valproate,<br>botulinum toxin	Improvement of tremor
**Lax et al, 2012[14] (n=1)**	NR	NR	NR	NR	NR	NR	NR
**Synofzik et al, 2012[15] (n=13)**	NR	NR	NR	NR	NR	NR	NR
**Woodbridge et al, 2013[16] (n=2)**	epilepsy (n=2)	no latency (n=1)<br>19 years (n=1)	elevated levels (n=2)	elevated protein levels (n=1)	multiple mtDNA deletions (n=1)	combination of AED (n=1)	• seizures (n=1)<br>• NR (n=1)
**Bereu et al, 2016[17] (n=15)**	NR	NR	NR	NR	NR	NR	NR
**Van Maldegem et al, 2016[18] (n=7)**	ataxia (n=5)<br>neuropathy (n=1)	no latency (n=5)<br>1 year (n=1)	NR	NR	multiple mtDNA deletions (n=4)<br>NR n=3	NR	• death (in less than 16 years) (n=4)<br>• alive (n=3)
**Janssen et al, 2016[19] (n=4)**	epilepsy (n=3)<br>ataxia (n=1)	0.5 years (n=1)<br>2 years (n=1)<br>3 years (n=1)<br>NR (n=1)	NR	NR	NR	valproate, lacozamide (n=1)<br>combination of AED (n=1)<br>NR (n=2)	• death (n=2)<br>• improvement of seizures (n=1)<br>• no seizures (n=1)
**Nicastro et al, 2016[20] (n=1)**	ataxia	no latency	NR	normal levels	NR	NR	wheelchair-bound within 4 years
**Henao et al, 2016[21] (n=1)**	ataxia	no latency	NR	NR	NR	NR	NR
**Paucar et al, 2016[22] (n=1)**	ataxia	NR	NR	NR	variation of fiber size and lack of COX activity in some	NR	NR

**Mongin et al, 2016[23] (n=1)**	ataxia	no latency	NR	NR	NR	NR	NR
**Rossi et al, 2017[24] (n=1)**	dystonia	no latency	normal levels	NR	NR	coenzyme Q10, flavonoids, creatine, and vitamins C and E; trihexyphenidyl for dystonia, levodopa therapy up to 1,000 mg/day	no improvement
**Vogel et al, 2017[25] (n=12)**	ataxia (n=10)<br>CPEO (n=1)<br>hypacusis (n=1)	no latency (n=10)<br>8 years (n=1)<br>14 years (n=1)	NR	NR	NR	NR	NR
**Jerath et al, 2018[26] (n=1)**	epilepsy	40 years	normal levels	NR	NR	topiramate, phenytoin, valproic acid	ataxia
**Piekutowska-Abramczuk et al, 2019[27] (n=2)**	ataxia	no latency (n=1)<br>NR (n=1)	NR	NR	multiple deletions	NR	NR
**Dosekova et al, 2020[28] (n=1)**	headache	10 years	NR	NR	NR	NR	NR
**Hikmat et al, 2020[29] (n=87/155)**	headache (73%)<br>seizure (69%)<br>ataxia (62%)<br>hypacusis (56%)	NR	elevated levels in serum (35%)	elevated protein levels (40%)	presence of ragged-red fibers, COX-negative fibers, and abnormal respiratory chain activities in fewer than the half of those investigated	NR	• Alive at the time of analysis (n=61/155)<br>• median age of death 7.4 years (1 month-91 years)<br>• cause of death: liver failure (32%, n = 30/93), infection/sepsis (20%, n = 19/93), multiorgan failure (19%, n = 18/93), status epilepticus (14%, n = 13/93), suicide (<0.1%, n=1), unknown (13%, n = 12/93)
**Bender et al, 2021[30] (n=24)**	ataxia (n=18)<br>epilepsy (n=3)<br>chorea (n=1)<br>CPEO (n=1)<br>hypacusis (n=1)	no latency (n=19)<br>3 years (n=1)<br>5 years (n=1)<br>7 years (n=1)<br>17 years (n=1)<br>21 years (n=1)	NR	NR	NR	NR	• loss of independent walking (n=6)<br>• wheelchair-bound (n=9)<br>• ataxia (n=4)<br>• death (n=4)<br>• NR (n=1)
**Nuzhnyi et al, 2021[31] (n=11)**	ataxia (n=5)<br>CPEO (n=3)<br>dysarthria (n=2)<br>epilepsy (n=1)	NR	NR	NR	NR	NR	NR
**Radziwonik et al, 2022[32] (n=4)**	NR	NR	NR	NR	NR	NR	NR
**Santos et al, 2022[33] (n=1)**	ataxia	no latency	NR	NR	NR	NR	NR

**Borsche et al, 2024[34] (n=2)**	n=2 ataxia	no latency	NR	NR	NR	NR	NR
**Smith et al, 2025[35] (n=5)**	NR	NR	NR	NR	NR	NR	death (between 46 and 79 years old) (n=5)

Abbreviations: n=number of patients with POLG mutation and ataxia, CSF: cerebrospinal fluid, AED: anti-epileptic drugs; CPEO: chronic progressive external ophthalmoplegia, NR: not reported; bid: two times a day, tid: three times a day, qid: four times a day.

Supplementary table *2*: Initial symptoms, laboratory findings and outcome of patients in studies of POLG-associated cerebellar ataxia

1. Van Goethem G, Luoma P, Rantamaki M, Al Memar A, Kaakkola S, Hackman P, et al. POLG mutations in neurodegenerative disorders with ataxia but no muscle involvement. *Neurology*. 2004;63(7):1251-7 DOI: 10.1212/01.wnl.0000140494.58732.83.
2. Winterthun S, Ferrari G, He L, Taylor RW, Zeviani M, Turnbull DM, et al. Autosomal recessive mitochondrial ataxic syndrome due to mitochondrial polymerase gamma mutations. *Neurology*. 2005;64(7):1204-8 DOI: 10.1212/01.WNL.0000156516.77696.5A.
3. Hakonen AH, Heiskanen S, Juvonen V, Lappalainen I, Luoma PT, Rantamaki M, et al. Mitochondrial DNA polymerase W748S mutation: a common cause of autosomal recessive ataxia with ancient European origin. *Am J Hum Genet*. 2005;77(3):430-41 DOI: 10.1086/444548.
4. Tzoulis C, Engelsen BA, Telstad W, Aasly J, Zeviani M, Winterthun S, et al. The spectrum of clinical disease caused by the A467T and W748S POLG mutations: a study of 26 cases. *Brain*. 2006;129(Pt 7):1685-92 DOI: 10.1093/braiNRwl097.
5. Galassi G, Lamantea E, Invernizzi F, Tavani F, Pisano I, Ferrero I, et al. Additive effects of POLG1 and ANT1 mutations in a complex encephalomyopathy. *Neuromuscul Disord*. 2008;18(6):465-70 DOI: 10.1016/j.nmd.2008.03.013.
6. Harrower T, Stewart JD, Hudson G, Houlden H, Warner G, O'Donovan DG, et al. POLG1 mutations manifesting as autosomal recessive axonal Charcot-Marie-Tooth disease. *Arch Neurol*. 2008;65(1):133-6 DOI: 10.1001/archneurol.2007.4.
7. Paus S, Zsurka G, Baron M, Deschauer M, Bamberg C, Klockgether T, et al. Apraxia of lid opening mimicking ptosis in compound heterozygosity for A467T and W748S POLG1 mutations. *Mov Disord*. 2008;23(9):1286-8 DOI: 10.1002/mds.22135.
8. Schulte C, Synofzik M, Gasser T, Schols L. Ataxia with ophthalmoplegia or sensory neuropathy is frequently caused by POLG mutations. *Neurology*. 2009;73(11):898-900 DOI: 10.1212/WNL.0b013e3181b78488.
9. Schicks J, Synofzik M, Schulte C, Schols L. POLG, but not PEO1, is a frequent cause of cerebellar ataxia in Central Europe. *Mov Disord*. 2010;25(15):2678-82 DOI: 10.1002/mds.23286.
10. Roshal D, Glosser D, Zangaladze A. Parieto-occipital lobe epilepsy caused by a POLG1 compound heterozygous A467T/W748S genotype. *Epilepsy Behav*. 2011;21(2):206-10 DOI: 10.1016/j.yebeh.2011.03.003.

11. Verhoeven WM, Egger JI, Kremer BP, de Pont BJ, Marcelis CL. Recurrent major depression, ataxia, and cardiomyopathy: association with a novel POLG mutation? *Neuropsychiatr Dis Treat*. 2011;7:293-6 DOI: 10.2147/NDT.S20153.
12. Habek M, Barun B, Adamec I, Mitrovic Z, Ozretic D, Brinar VV. Early-onset ataxia with progressive external ophthalmoplegia associated with POLG mutation: autosomal recessive mitochondrial ataxic syndrome or SANDO? *Neurologist*. 2012;18(5):287-9 DOI: 10.1097/NRL.0b013e318266f5a6.
13. Hinnell C, Haider S, Delamont S, Clough C, Hadzic N, Samuel M. Dystonia in mitochondrial spinocerebellar ataxia and epilepsy syndrome associated with novel recessive POLG mutations. *Mov Disord*. 2012;27(1):162-3 DOI: 10.1002/mds.23960.
14. Lax NZ, Hepplewhite PD, Reeve AK, Nesbitt V, McFarland R, Jaros E, et al. Cerebellar ataxia in patients with mitochondrial DNA disease: a molecular clinicopathological study. *J Neuropathol Exp Neurol*. 2012;71(2):148-61 DOI: 10.1097/NEN.0b013e318244477d.
15. Synofzik M, Srulijes K, Godau J, Berg D, Schols L. Characterizing POLG ataxia: clinics, electrophysiology and imaging. *Cerebellum*. 2012;11(4):1002-11 DOI: 10.1007/s12311-012-0378-2.
16. Woodbridge P, Liang C, Davis RL, Vandebona H, Sue CM. POLG mutations in Australian patients with mitochondrial disease. *Intern Med J*. 2013;43(2):150-6 DOI: 10.1111/j.1445-5994.2012.02847.x.
17. Bereau M, Anheim M, Echaniz-Laguna A, Magot A, Verny C, Goideau-Sevrain M, et al. The wide POLG-related spectrum: An integrated view. *J Neurol Sci*. 2016;368:70-6 DOI: 10.1016/j.jns.2016.06.062.
18. Van Maldergem L, Besse A, De Paepe B, Blakely EL, Appadurai V, Humble MM, et al. POLG2 deficiency causes adult-onset syndromic sensory neuropathy, ataxia and parkinsonism. *Ann Clin Transl Neurol*. 2017;4(1):4-14 DOI: 10.1002/acn3.361.
19. Janssen W, Quaegebeur A, Van Goethem G, Ann L, Smets K, Vandenberghe R, et al. The spectrum of epilepsy caused by POLG mutations. *Acta Neurol Belg*. 2016;116(1):17-25 DOI: 10.1007/s13760-015-0499-8.
20. Nicastro N, Ranza E, Antonarakis SE, Horvath J. Pure Progressive Ataxia and Palatal Tremor (PAPT) Associated with a New Polymerase Gamma (POLG) Mutation. *Cerebellum*. 2016;15(6):829-31 DOI: 10.1007/s12311-015-0749-6.
21. Henao AI, Pira S, Herrera DA, Vargas SA, Montoya J, Castillo M. Characteristic brain MRI findings in ataxia-neuropathy spectrum related to POLG mutation. *Neuroradiol J*. 2016;29(1):46-8 DOI: 10.1177/1971400915621324.
22. Paucar M, Engvall M, Gordon L, Tham E, Synofzik M, Svenningsson P. POLG-Associated Ataxia Presenting as a Fragile X Tremor/Ataxia Phenocopy Syndrome. *Cerebellum*. 2016;15(5):632-5 DOI: 10.1007/s12311-016-0777-x.
23. Mongin M, Delorme C, Lenglet T, Jardel C, Vignal C, Roze E. Progressive Ataxia and Palatal Tremor: Think about POLG Mutations. *Tremor Other Hyperkinet Mov (N Y)*. 2016;6:382 DOI: 10.7916/D86M36RK.
24. Rossi M, Medina Escobar A, Radrizzani M, Tenembaum S, Perandones C, Merello M. Dystonia in a Patient with Autosomal-Dominant Progressive External Ophthalmoplegia Type 1 Caused by Mutation in the POLG Gene. *Mov Disord Clin Pract*. 2017;4(2):266-9 DOI: 10.1002/mdc3.12397.
25. Vogel AP, Rommel N, Oettinger A, Horger M, Krumm P, Kraus EM, et al. Speech and swallowing abnormalities in adults with POLG associated ataxia (POLG-A). *Mitochondrion*. 2017;37:1-7 DOI: 10.1016/j.mito.2017.06.002.
26. Jerath NU, Shy ME. Asymmetric Ataxia, Depression, Memory Loss, Epilepsy, and Axonal Neuropathy Associated with A Heterozygous DNA Polymerase Gamma Variant of Uncertain Significance, c1370G>a (R457Q). *J Neuromuscul Dis*. 2018;5(1):99-104 DOI: 10.3233/JND-170229.

27. Piekutowska-Abramczuk D, Kaliszewska M, Sulek A, Jurkowska N, Oltarzewski M, Jablonska E, et al. The frequency of mitochondrial polymerase gamma related disorders in a large Polish population cohort. *Mitochondrion*. 2019;47:179-87 DOI: 10.1016/j.mito.2018.11.004.
28. Dosekova P, Dubiel A, Karlowicz A, Zietkiewicz S, Rydzanicz M, Habalova V, et al. Whole exome sequencing identifies a homozygous POLG2 missense variant in an adult patient presenting with optic atrophy, movement disorders, premature ovarian failure and mitochondrial DNA depletion. *Eur J Med Genet*. 2020;63(4):103821 DOI: 10.1016/j.ejmg.2019.103821.
29. Hikmat O, Naess K, Engvall M, Klingenberg C, Rasmussen M, Tallaksen CM, et al. Simplifying the clinical classification of polymerase gamma (POLG) disease based on age of onset; studies using a cohort of 155 cases. *J Inherit Metab Dis*. 2020;43(4):726-36 DOI: 10.1002/jimd.12211.
30. Bender F, Timmann D, van de Warrenburg BP, Adarmes-Gomez AD, Bender B, Thieme A, et al. Natural History of Polymerase Gamma-Related Ataxia. *Mov Disord*. 2021;36(11):2642-52 DOI: 10.1002/mds.28713.
31. Nuzhnyi E, Seliverstov Y, Klyushnikov S, Krylova T, Tsygankova P, Bychkov I, et al. POLG-associated ataxias can represent a substantial part of recessive and sporadic ataxias in adults. *Clin Neurol Neurosurg*. 2021;201:106462 DOI: 10.1016/j.clineuro.2020.106462.
32. Radziwonik W, Elert-Dobkowska E, Klimkowicz-Mrowiec A, Ziora-Jakutowicz K, Stepniak I, Zaremba J, et al. Application of a custom NGS gene panel revealed a high diagnostic utility for molecular testing of hereditary ataxias. *J Appl Genet*. 2022;63(3):513-25 DOI: 10.1007/s13353-022-00701-3.
33. Santos M, Damasio J, Carmona S, Neto JL, Dehghani N, Guedes LC, et al. Molecular Characterization of Portuguese Patients with Hereditary Cerebellar Ataxia. *Cells*. 2022;11(6) DOI: 10.3390/cells11060981.
34. Borsche M, Dulovic-Mahlow M, Baumann H, Tunc S, Luth T, Schaake S, et al. POLG2-Linked Mitochondrial Disease: Functional Insights from New Mutation Carriers and Review of the Literature. *Cerebellum*. 2024;23(2):479-88 DOI: 10.1007/s12311-023-01557-x.
35. Smith LA, Olkhova EA, Lax NZ, Ng YS, Taylor RW, Gorman GS, et al. Delineating the mechanisms of cerebellar degeneration in paediatric and adult primary mitochondrial disease. *Acta Neuropathol*. 2025;149(1):53 DOI: 10.1007/s00401-025-02891-6.
