# Supplementary material for: A Scoping Review of POLG-Related Cerebellar Ataxia: Insights and Clinical Perspectives
Source: Tremor Other Hyperkinet Mov (N Y). 2025 Nov 10;15:55. doi: 10.5334/tohm.1027 (PMC12617407; doi:10.5334/tohm.1027)
Supplement: Supplementary Table 3. — Diagnostic clues to distinguish POLG-related cerebellar ataxia from other common ataxias. [file tohm-15-1-1027-s3.pdf]

	POLG-related ataxia	Friedreich's ataxia	Spinocerebellar ataxias	Immune-mediated ataxias (gluten-related, GAD)	Multiple system atrophy
**Age of onset**	varies greatly (childhood-late adulthood)	<25 years	usually >25 years (childhood-70s)	40s-50s	>60 years
**Peripheral neuropathy**	yes	yes	in some types (esp. SCA 1,2,3,4, 43, 46)	frequent (gluten-related)	no
**CPEO**	yes	no	in some types (esp. SCA 2, 3, 28, 40)	no	no
**Epilepsy**	yes	no	in some types (SCA 10, 19, 22)	frequent (GAD, gluten-related, SLE)	no
**Eye findings**	nystagmus not common	fixation instability, saccadic dysmetria, disrupted pursuit, square-wave jerks	gaze-evoked, vertical nystagmus	not common, gaze-evoked	gaze-evoked horizontal, downbeat nystagmus, saccadic hypermetria
**Other movement disorders**	myoclonus, tremor	not common	parkinsonism (SCA 2, 3, 10, 14, 17, 19, 21), tremor (SCA 2, 12, 15, 27), dystonia (SCA 3, 14, 17, 20, 35)	RLS, myoclonus (gluten-related)	parkinsonism
**Other clinical features**	hearing loss, migraine, cognitive impairment, affective symptoms	pyramidal signs, pes cavus, scoliosis, diabetes, cardiomyopathy	pyramidal signs, cognitive or affective symptoms, dysautonomia (esp. SCA 1,2,3,4)	gastrointestinal (gluten-related, GAD) diabetes, thyroid disease, vitiligo (GAD)	dysautonomia, RBD
**Disease progression (loss of independent walking)**	over decades	in 10-15 years	over 10-20 years (depending on type, repeat expansion)	over months/years	rapid
**Imaging findings**	cerebellar atrophy, cerebellar and olivary nucleus changes	no cerebellar atrophy	cerebellar, brainstem, spinal cord atrophy	cerebellar atrophy in 50%	cerebellar atrophy, ''hot-cross'' bun sign
**Laboratory findings**	increased lactate in serum and/or CSF, muscle biopsy with ragged red fibers, COX-deficient fibers, and/or multiple mtDNA deletions	ECG changes	not typical	anti-GAD antibodies (GAD ataxia), gliadin and transglutaminase antibodies (gluten-related)	not typical

Supplementary table 3. Diagnostic clues to distinguish POLG-related ataxia from other common ataxias. Abbreviations: esp.=especially, GAD= glutamic acid decarboxylase, SLE= systematic lupus erythematosus, CPEO=chronic progressive ophthalmoplegia, SCAs=spinocerebellar ataxias, RLS= restless legs syndrome, RBD=REM-sleep behavior disorder, ECG=electrocardiogram, based on References [1-7]

1. Reetz K, Lischewski SA, Dogan I, Didszun C, Pishnamaz M, Konrad K, Marx-Schütt K, Farmer J, Lynch DR, Corben LA *et al*: **Friedreich's ataxia-a rare multisystem disease**. *The Lancet Neurology* 2025, **24**(7):614-624.
2. Sullivan R, Yau WY, O'Connor E, Houlden H: **Spinocerebellar ataxia: an update**. *Journal of neurology* 2019, **266**(2):533-544.
3. Stankovic I, Fanciulli A, Sidoroff V, Wenning GK: **A Review on the Clinical Diagnosis of Multiple System Atrophy**. *Cerebellum* 2023, **22**(5):825-839.
4. Hadjivassiliou M, Sanders DD, Aeschlimann DP: **Gluten-related disorders: gluten ataxia**. *Digestive diseases (Basel, Switzerland)* 2015, **33**(2):264-268.
5. Baizabal-Carvallo JF, Alonso-Juarez M: **Cerebellar disease associated with anti-glutamic acid decarboxylase antibodies: review**. *Journal of neural transmission* 2017, **124**(10):1171-1182.
6. Vinagre-Aragón A, Zis P, Grunewald RA, Hadjivassiliou M: **Movement Disorders Related to Gluten Sensitivity: A Systematic Review**. *Nutrients* 2018, **10**(8).
7. Lin CR, Kuo SH, Opal P: **Cognitive, Emotional, and Other Non-motor Symptoms of Spinocerebellar Ataxias**. *Current neurology and neuroscience reports* 2024, **24**(3):47-54.
